# Effects of increase in temperature and open water on transmigration and access to health care by the Nenets reindeer herders in northern Russia

**DOI:** 10.3402/ijch.v72i0.21183

**Published:** 2013-08-05

**Authors:** Philippe Amstislavski, Leonid Zubov, Herman Chen, Pietro Ceccato, Jean-Francois Pekel, Jeremy Weedon

**Affiliations:** 1 Department of Occupational and Environmental Health Sciences, School of Public Health, State University of New York Downstate Medical Center, Brooklyn, New York, USA; 2 Northern Medical University, Arkhangelsk, Russia; 3 The International Research Institute for Climate and Society, The Earth Institute, Columbia University, Palisades, New York, USA; 4 Joint Research Center, European Commission, Ispra, Varese, Italy

**Keywords:** climate change, health services access, hydrology, indigenous peoples, permafrost, Rangifer tarandus, remote sensing, satellite imagery, surface water, water bodies

## Abstract

**Background:**

The indigenous Nenets reindeer herders in northern Russia annually migrate several hundred kilometers between summer and winter pastures. In the warming climate, ice-rich permafrost and glaciers are being significantly reduced and will eventually disappear from parts of the Arctic. The emergent changes in hydrological cycles have already led to substantial increases in open water that stays unfrozen for longer periods of time. This environmental change has been reported to compromise the nomadic Nenets’ traditional way of life because the presence of new water in the tundra reduces the Nenets’ ability to travel by foot, sled, or motor vehicle from the summer transitory tundra campsites in order to access healthcare centers in villages. New water can also impede their access to family and community at other herder camps and in the villages. Although regional and global models predicting hydrologic changes due to climate changes exist, the spatial resolution of these models is too coarse for studying how increases in open water affect health and livelihoods. To anticipate the full health impact of hydrologic changes, the current gap between globally forecasted scenarios and locally forecasted hydrologic scenarios needs to be bridged.

**Objectives:**

We studied the effects of the autumn temperature anomalies and increases in open water on health care access and transmigration of reindeer herders on the Kanin Peninsula.

**Design:**

Correlational and time series analyses were completed.

**Methods:**

The study population consisted of 370 full-time, nomadic reindeer herders. We utilized clinical visit records, studied surface temperature anomalies during autumn migrations, and used remotely sensed imagery to detect water bodies. Spearman correlation was used to measure the relationship between temperature anomalies and the annual arrival of the herders at the Nes clinic for preventive and primary care. Piecewise regression was used to model change in mean autumnal temperature anomalies over time. We also created a water body product to detect inter-annual changes in water area.

**Results:**

Correlation between arrivals to the Nes clinic and temperature anomalies during the fall transmigration (1979–2011) was r = 0.64, p = 0.0004; 95% CI (0.31; 0.82). Regression analysis estimated that mean temperature anomalies during the fall migration in September–December were stochastically stationary pre-1991 and have been rising significantly (p < 0.001) since then. The rate of change was estimated at +0.1351°C/year, SE = 0.0328, 95% CI (+0.0694, +0.2007). The amount of detected water fluctuated significantly interannually (620–800 km^2^).

**Conclusions:**

Later arrival of freezing temperatures in the autumn followed by the earlier spring thaws and more open water delay transmigration and reduce herders’ access to health care. The recently observed delays in arrival to the clinic are likely related to the warming trend and to concomitant hydrologic changes.

Permafrost temperature has increased by 1°C to 2°C in the Russian north during the last 3 decades (1). Northern Alaska and northern Canada also have warmed to a comparably high level during the same period (2). Recent observations indicate that this warming is highly coupled with a shortened winter season and the degradation of the ice-rich permafrost (3–5). In the Arctic, these impacts are profoundly affecting communities because even relatively small landscape changes due to warming have been shown to impact the fragile tundra socio-ecological systems out of scale with their spatial extent (5,6).

Most terrestrial and aquatic components of the Russian tundra are seasonally exploited by the indigenous nomadic reindeer herders, hunters, and fishers (5). Because of the high level of interdependence between the human and environmental conditions and poor access to health services in the Russian far north, rapid shifts in hydrologic cycles in the tundra are expected to have far-reaching consequences on health and the social fabric of the local indigenous communities (7).

Across the Russian north, non-emergency medical care is provided through a sparse network of small, village-based health clinics. Nomadic Nenets use the traditional reindeer sled, walking, and increasingly, the snowmobile, as their means of transportation across vast distances. They migrate several hundred kilometers with large reindeer herds in the spring and autumn. The migration paths connect the summer pastures on the nutrient-rich grassy tundra zone along the Arctic coast and the winter pastures within the forest-tundra zone. During these migrations, the nomadic reindeer herders pass through the same villages that are located on the long-established annual migration routes. Non-emergency visits to the health clinic typically take place during these transmigrations, when the herder families receive maternal, preventative, and primary care. It is reasonable to assume that the appearance of new lakes, marshland, and streams as well as a rapid change in the size of the existing water bodies is likely to pose new geographic barriers that may reduce or preclude the herders’ access to the already sparse, remote, and often difficult to reach preventive and health care services.

According to 1 study of Nenets herders, the main effects of the recent warming are increases in open water in the tundra and frequent, unusually warm, autumns (8). These events create transportation conditions that are difficult and unpredictable (5). In the summer months, when the herders are in the coastal tundra, they are the furthest from medical help. The appearance of a new stream in the previously dry valley from 1 year to another can block the most direct route to the village and its clinic and make travel there exceedingly difficult. In some cases, this could add several days of travel on new and unfamiliar terrain to an already long journey to reach medical care. Fall-through-ice events may occur along these new routes and may present additional health hazards. However, the data on these incidents was not available at the time this article was being written.

Traditionally, autumn transmigration began with the arrival of stable sub-freezing temperatures, which establish firm ice on the fresh water bodies allowing the herders and thousands of reindeer to cross the rivers and lakes. The recent rapid warming created delayed formation of stable ice and concomitant setbacks for herders in leaving remote coastal pastures and in accessing health clinics. It also may extend the herders’ stay in the grassy tundra pastures on the Arctic coast, often hundreds of kilometers from the closest settlement. In the Arctic, where geographic access to kin and communities of other herders are critical for survival, ability to reach other herders, scattered across dozens of camps on the vast tundra, is also affected by the nascent hydrological and environmental changes. For example, when footpaths that once linked age-old camps of different families become inundated the herders cannot access other camps by foot or sled.

Other problems related to the recent hydrologic changes reported by Nenets herders include loss of the valuable reindeer grazing and calving habitats to newly appeared lakes and marshland, loss of the long-established campsites and traditional migratory routes, and loss of the sacred religious sites and burial grounds to inundation (9). Reported problems securing food include impeded access to wild berries and mushrooms and to hunting and fishing areas; these problems may affect the nutritional status of the herders (8). Securing food is critical for nomadic Nenets because reduced access to traditional food forces a greater reliance on non-traditional food that is only available in the distant villages, which in turn become less geographically accessible due to the changing, wetter landscape (10).

## Nomadic Nenets and reindeer husbandry in the Russian Arctic

**Fig. 1:**
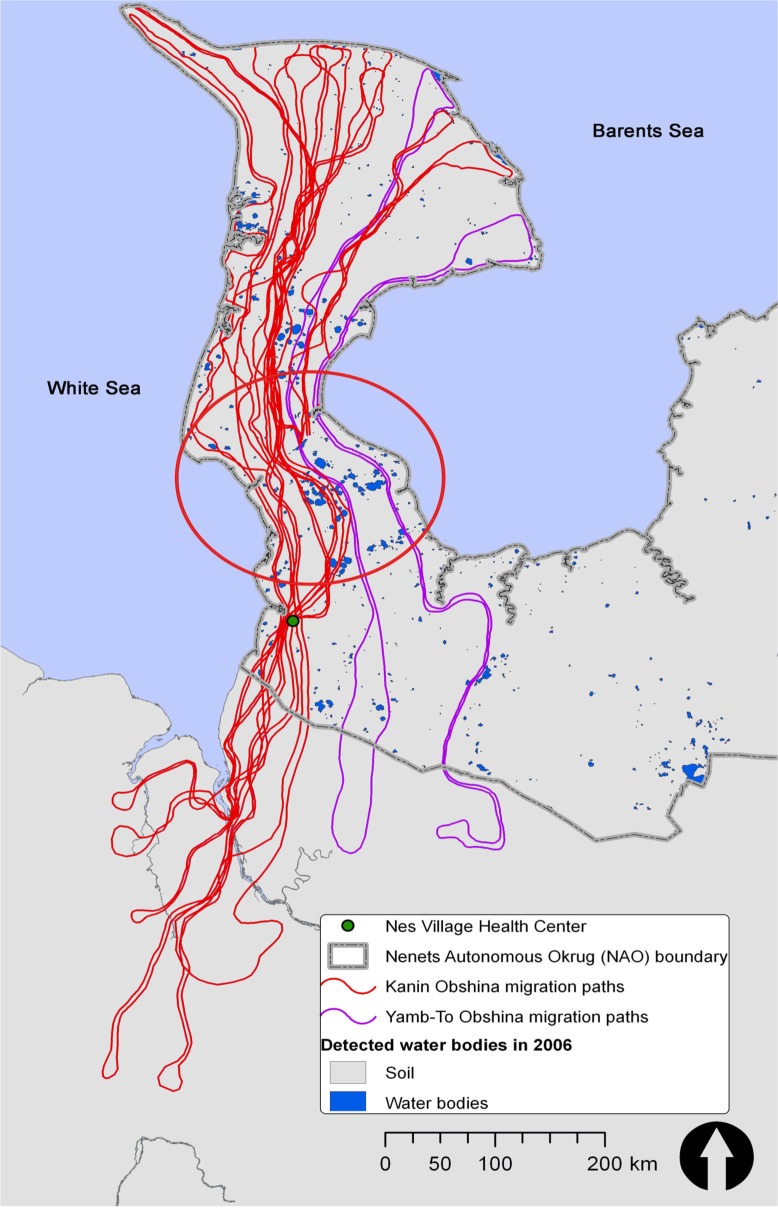
Kanin and Yamb-To Obshina migratory routes connecting the summer pastures on Kanin Peninsula to the winter camps in lower-latitude areas. Nes serves as a key logistics and health care hub for most herders in the Kanin area. The cluster of lakes and marshland at Kanin Neck is highlighted by the red oval.

Nenets are indigenous Arctic people living predominantly along the Arctic Ocean in northwest Russia, and many still practice reindeer herding and the nomadic way of life. The nomadic Nenets and their predecessors began hunting and harnessing reindeer during migrations between 500 and 1100 AD (11); herding became progressively more intensive after 1600 AD (12). Nenets retain a unique culture, which strongly emphasizes their ties to the reindeer, the tundra, and to the nomadism, with stewardship of the tundra ecosystem playing a key role. In the Nenets Autonomous Okrug (NAO), families engaged in full-time reindeer herding annually migrate up to 1,200 km with their herds between the grassy summer pastures on the coast and the winter pastures in the forest tundra south of the NAO and in the Arkhangelsk Oblast (8–10). The NAO (Fig. 1) is home to the second largest Nenets populations in the world (13). In 2008, the NAO (176,700 km^2^) was home to 5,623 Nenets, who constituted 13.5% of the total population of the Okrug (8).

Reindeer (*Rangifer tarandus* L.) are important nutritionally, economically, and socio-culturally within the tundra socioecological system (SES). Reindeer husbandry is the key traditional occupation and a way of life for Nenets in the NAO where the *Rangifer tarandus* population is the second largest in Russia (9). There were 157,000 domesticated reindeer in the NAO in 2007, and Kanin Peninsula in the far northwestern NAO had the largest reindeer population in the Okrug (8), providing the main source of protein to the herder familes, as well as skins and income from sales of surplus deer meat. The total territory of reindeer pastures is 132,000 km^2^, or 75% of NAO land area (8,9). There are some 20 indigenous reindeer herder groups and the number of individuals employed in reindeer husbandry recently grew from 818 (2003) to 1,100 (2007). Approximately, two-thirds of the NAO's herders still lead nomadic lives.

## Reindeer husbandry on Kanin Peninsula

Our study site is Kanin Peninsula (14,673 km^2^), located in the extreme northwestern NAO—surrounded by the White Sea to the west and the Barents Sea to the north and east. Kanin is relatively flat, undulating, low-laying grassy tundra, punctuated by many small lakes and vast expenses of marshland, rivers, and streams that are deeply cut into the surface and not crossable during the summer months. The southern part of the peninsula is called Kanin Neck. This area is especially narrow and is composed of low-lying lakes and marshland, and thus is largely impassable in the summer. During transmigrations, when herders travel with thousands of reindeer and loaded sleds, they can only cross in and out of Kanin Peninsula when the open water on Kanin Neck is frozen, which creates an objective measure of the warming's effect on the timing of transmigration and on arrivals to the clinic.

Unlike some of the other parts of the NAO, Kanin has not been affected by the recent hydrocarbon development boom in the region and the local population remains very small, with 5 villages and the only full-time health clinic and hospital in Nes (2007 pop., 1,256). During the Soviet era, the herders were organized into Obshini (communities) with non-overlapping calving and grazing territories for their herds, and this structure is still largely in place. On Kanin Peninsula, there are 2 herder communities, Kanin Obshina (370 members) and Yamb-To Obshina (230 members) each comprising of smaller, kin-based herder groups (8,9). The Kanin Obshina community's territory is along the White Sea coast of the peninsula and the Yamb-To territory is on the eastern side of the peninsula along the Barents Sea.

During the spring and fall migrations, Kanin Obshina herders pass through the village of Nes where the health clinic is located, while the Yamb-To Obshina pass through the village of Vizas. The location of Nes, on the traditional migration paths of the Kanin Obshina just south of Kanin Neck, makes it an important hub for herders—where they stop to resupply and to seek health care during spring and autumn migrations (Fig. 1).

The local health care providers and the tundra residents have reported that temperature change and shorter, warmer winters have led to the expansion of the bodies of water, which causes hitherto unseen changes in the landscape and delays migration along the traditional routes travelled by generations of reindeer herders during the annual migrations (8). Since 1979, dates have been recorded for the herders’ fall arrival at the Nes clinic during their transmigration south and for some of the spring arrivals during their transmigration north. These dates provide useful information on the chorology of the change in tundra conditions with respect to health care access.

The purpose of this research is to quantify the effects of temperature anomalies and of increases in open water on transmigration and health care access by Nenets reindeer herders in northern Russia.

## Materials and methods

**Fig. 2:**
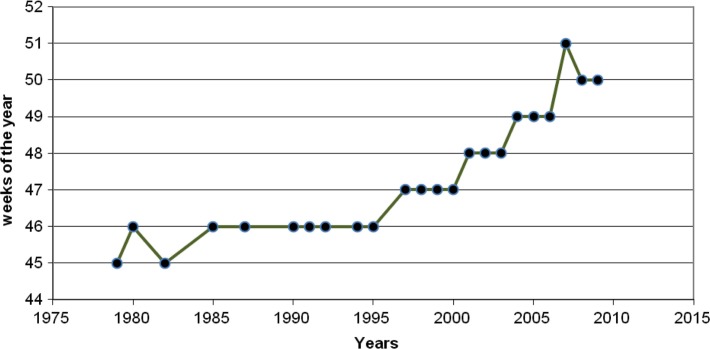
Arrivals of Kanin herders at the Nes Health Center.

We hypothesized that a pronounced increase in temperature will have an impact on access to health care in the Arctic due to its effect on transportation and on other aspects of nomadic herder livelihoods. The raising temperatures are most likely to affect access due to new barriers to transmigration and to visits to the Nes health clinic, namely because of a shorter winter season and more open water. We employed clinical records on dates of arrivals in Nes for the reindeer herders during the fall migrations from 1979–2011 (Fig. 2); these were collected by one of the authors (L. Zubov). We also used a water bodies mask derived from daily surface reflectance images produced by moderate resolution imaging spectroradiometer (MODIS) from 2004–2011 in order to calculate the total territory of the open water in the tundra.

We used the mean of the temperature anomalies (14) for the 4 autumn migration months for quantitative analysis of the relationship of interannual autumn temperature anomalies trends and autumn arrivals to the Nes clinic.

### Remotely sensed water bodies data

The main challenge in identifying water surfaces from remotely sensed data is the high variability of their spectral signatures. The spectral property of water is determined by the electromagnetic interaction of light with the constituent components of water via absorption or scattering processes either within the water column, at the water surface, or on the bottom of the body of water (15). Consequently, the water-leaving radiance detected by the sensor shows great spatial and temporal variability, which makes its reliable discrimination particularly difficult (16).

In the framework of this study, we used a methodology proposed by Pekel et al. in 2012 to detect open water surfaces in near real time. The method is based on a colorimetric approach. The rationale we employed is that the perceived color in a color composition is directly determined by the shape of the spectral signature of the signal's target. Our novel approach permitted us to associate both land and water surfaces with unique colors. We identified a set of thresholds to detect the open water surfaces on a 10-day basis at a resolution of 250 meters.

Some false water surface detections were manifested by a low temporal frequency. These false detections resulted from the remaining perturbations after atmospheric correction and cloud removal. Indeed, over our area of interest, the MODIS standard flags were not masking all the contaminated values; these were then included in the compositing process and induced reflectance instability and consequently some false detection. In order to discard these false detections from our analysis, we considered only the water surfaces showing an occurrence (i.e. number of water detections divided by the number of available observations) above 35% on an annual basis. Indeed, because the false detections occurred randomly, both spatially and temporally with a low temporal frequency, the lower occurrence concentrated the false detections. One drawback of this approach is that temporary water surfaces also characterized by low annual occurrence were discarded. Obviously, it decreased artificially the number of water surfaces considered in our study but because the same criterion were applied for each year, and because we used these detections in a relative way, it did not significantly impact our results. For this research, the new water body mask products were created for the period 2004 to 2009 and were mapped. An operational open water detection product from 2001 to the present will be made available in the near future.

**Fig. 3:**
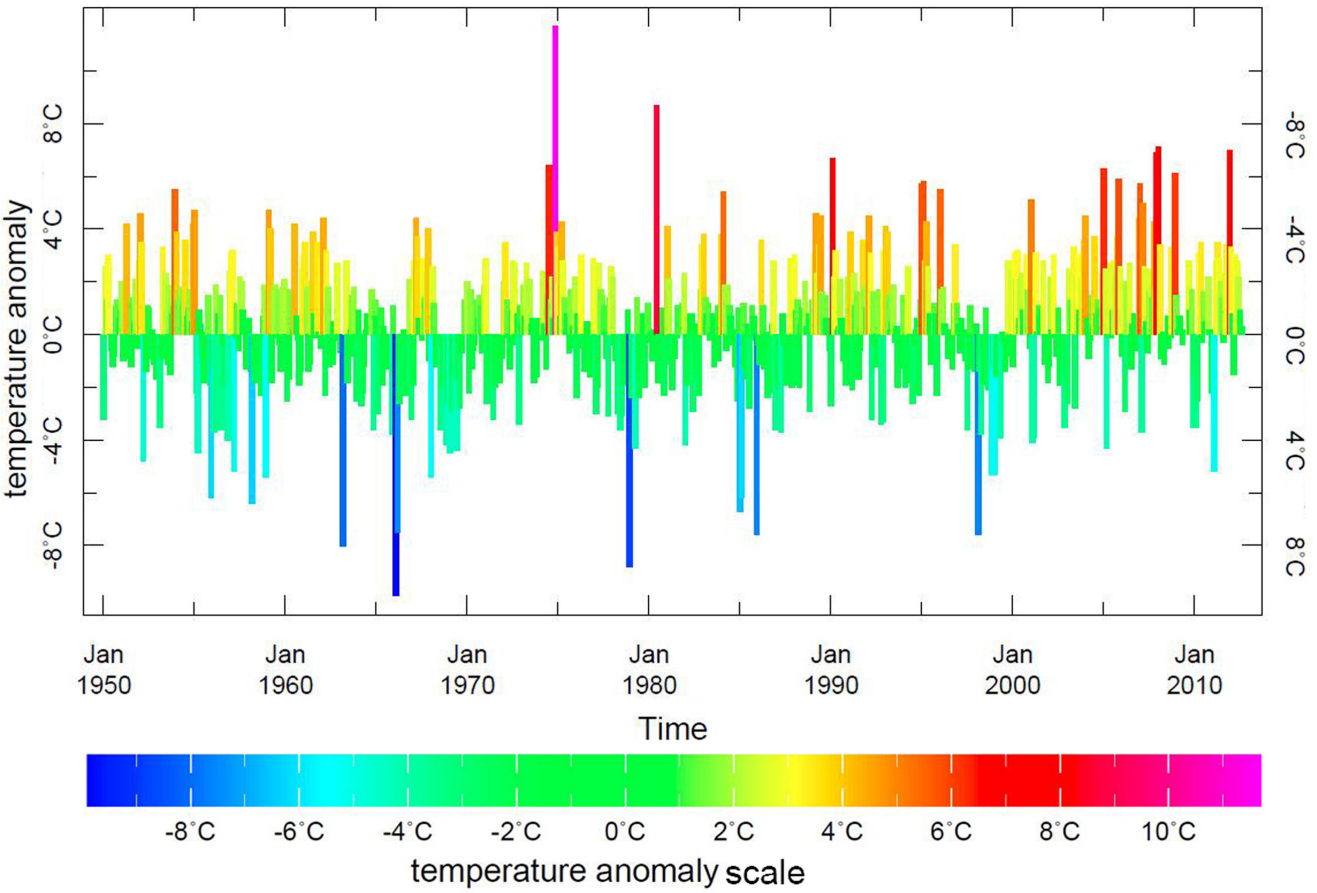
Monthly anomalies in air temperatures at 1.0 degree spatial resolution from the Climate Anomaly Monitoring System (CAMS) produced by the National Oceanic and Atmospheric Administration’s Climate Prediction Center.

### Temperature anomaly data

To assess changes in temperature trends, we used monthly anomalies in air temperature data at 1.0 degree spatial resolution from the Climate Anomaly Monitoring System (CAMS) monthly gridded and station data produced by the National Oceanic and Atmospheric Administration's Climate Prediction Center (16), shown in Fig. 3. In the world of climate studies, the term “anomaly” means the difference between the value of a quantity and its climatological mean value. A “monthly anomaly” is the difference between the original monthly value of a quantity in a given month and the monthly climatological value for that month of the year. The monthly temperature anomaly equation is written as

1 $${r^{\prime }}_{ij}=r_{ij}-\frac{1}{N_{i}}\sum_{j=1}^{N}r_{ij}$$

Here, for months *i* and years *j*, *r′*_*ij*_ is the monthly temperature anomaly, *r*_*ij*_ is the original monthly value, and the remainder of the equation is the calculation of the monthly temperature (which is subtracted from the original monthly values). For CAMS, the long-term average is computed from 1950 to the present.

**Fig. 4:**
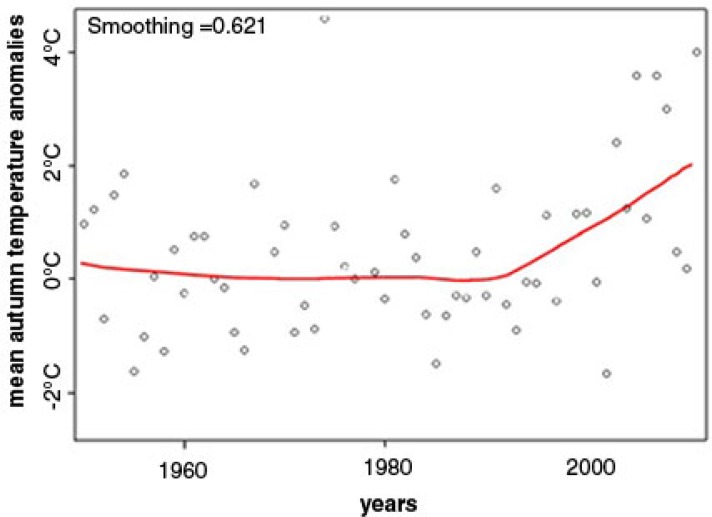
Temperature anomalies trend data for the Kanin Peninsula from the CAMS database for 1950–2012 showing the recent increase in positive anomalies and the LOESS plot of the autumnal temperature anomalies for the same period. The LOESS plot strongly suggests a relationship between calendar year and mean autumnal temperature anomalies as having 2 linear components: one relevant to the years 1950–1991, the other to the post-1991 period, when the temperature began to raise sharply.

For the Kanin area, we computed a monthly temperature anomaly average over a territory that covers the entire study area. The data were extracted from the CAMS data set using the Ingrid code. The CAMS data set is located at the International Research Institute for Climate and Society and the Lamont-Doherty Earth Observatory (IRI/LDEO) Climate Data Library at Columbia University and available at: http://iridl.ldeo.columbia.edu (Fig. 4).

### Data analyses and statistical methods

To assess changes over time in herd movement, a Spearman correlation between calendar year (1979–2011) and week of arrival at Nes village was computed, along with a 95% confidence interval; parametric analysis was not attempted due to sparsity of data. To assess change over time in autumn migration season (September–December) temperature for the years 1950–2011, a local regression or LOESS plot was generated that strongly suggested a relationship between calendar year and mean temperature as having two linear components: one relevant to the years 1950–1991, the other to the post-1991 period (Fig. 4). LOESS is a strategy for fitting smooth curves to empirical bivariate data for visualization and further time series analysis. The LOESS statistical procedure is a fairly direct generalization of traditional least-squares methods for data analysis.

A mixed linear model was therefore constructed, with mean temperature for September–December as the dependent variable, calendar year as the predictor, modeled as a continuous piecewise linear relationship with a slope cut point at 1991 (in other words, as a linear spline with a single predetermined knot). First order autoregressive moving-average (ARMA (1,1)) structure was used to model autocorellation. Model residuals suggested 1974 to be an outlier year; results presented here exclude data for that year. ArcGIS 10 (ESRI, Redlands, California) software was used for mapping and SAS Release 9.2 (SAS Institute, Cary, North Carolina) software was used for statistical analysis.

## Results

Figure 4 displays the CAMS temperature anomalies data for Kanin for the years 1950–2012 and the LOESS plot of the autumnal anomalies for the same period. Temperature anomalies data show the recent increase in positive anomalies throughout the year. The LOESS plot strongly suggests an increase in more recent mean autumnal temperature anomalies (September–December) after 1991 as having 2 linear components: one relevant to the years 1950–1991, the other to the post-1991 period, when the temperature began to raise sharply.

Correlation of the field-collected data on the herders’ fall arrival at Nes and the calendar year for the last 32 years produced r = 0.64, p-value of <0.001, and 95% CI (0.31; 0.82). Regression analysis estimated that mean temperature anomalies during the fall migration in September–December were stochastically stationary pre-1991 (−0.0157 Cdeg/year, SE = 0.0140, 95% CI [−0.0437, +0.0122], p = 0.265) but have risen significantly (p < 0.001) since then. The rate of change is estimated at 0.7 to 2.0 Cdeg/decade +0.1351 Cdeg/year, SE = 0.0328, 95% CI (+0.0694, +0.2007); test of slope change at 1991: p = 0.001.

The amount of detected open water area and the number of water bodies per year (2004–2009) have fluctuated significantly within the study area on Kanin Peninsula—from approximately 600 km^2^ to more than 800 km^2^.

This illustrates the current instability of the total water area within the migration range of the herders. Specifically, in some years (e.g. 2004), a rapid increase in open water area is followed by a sharp drop the next year, indicating rapid draining of many water bodies. More research is needed to better understand these novel biophysical processes and their future implications on the well-being of Arctic social and ecological systems.

**Fig. 5:**
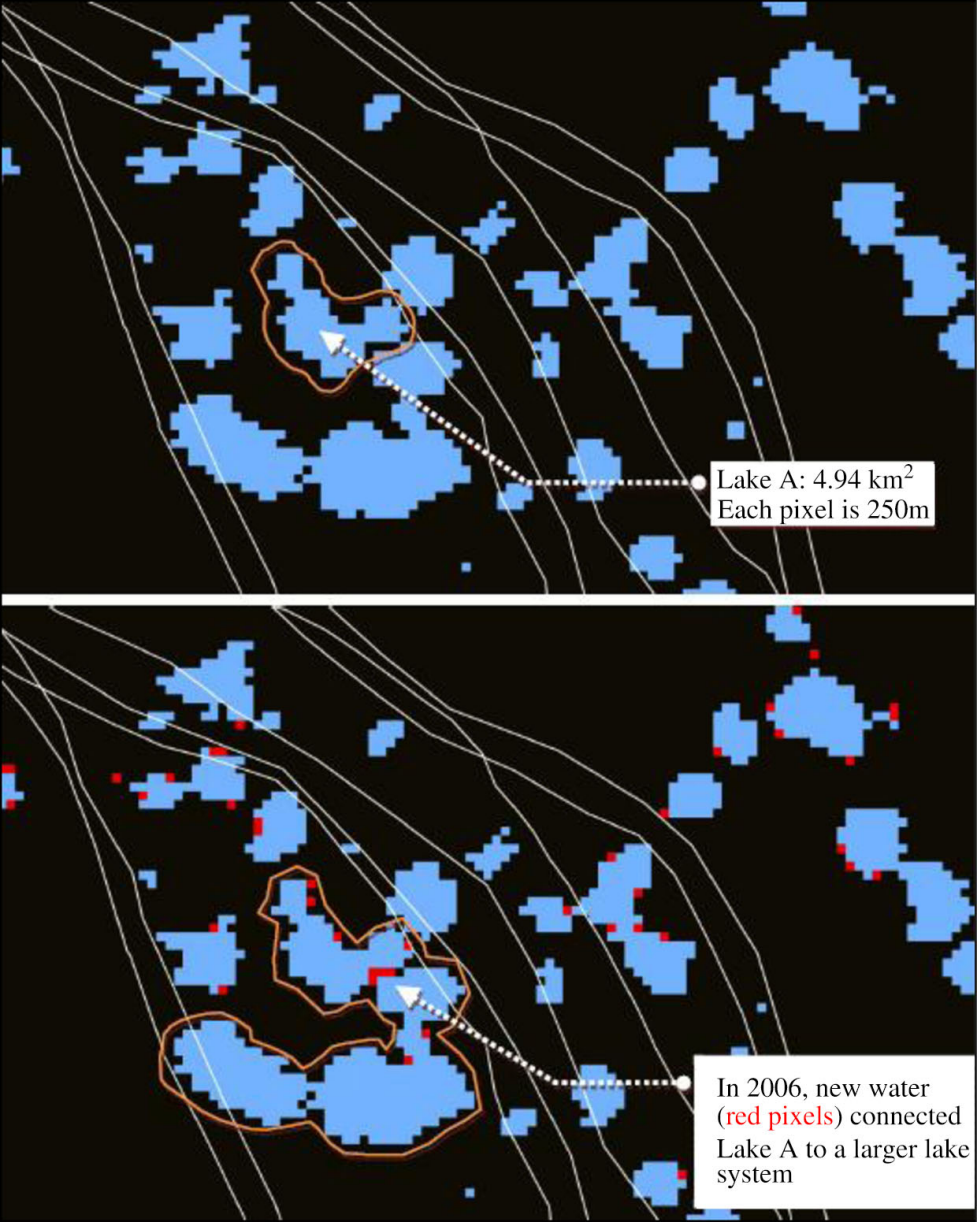
Effect of increase in surface water in the study area. The top image shows water areas detected in 2005. Blue pixels indicate existing open water. White lines indicate approximate locations of historic migration paths used by the herders.

Visual inspection of the overlays of the annual water masks with the transmigration routes in a geographic information system or GIS revealed that many newly detected water bodies lay directly on the routes. We examined one such area on Kanin Neck (shown in Fig. 5), where large interannual changes in open water were detected by our method, and noted that there—and in many low areas on Kanin—even a modest increase in open water area caused several lakes to merge into single large bodies of water, thereby creating impassible barriers to ground transportation. In Fig. 5, Lake A has increased from 4.94 km^2^ in 2005 to 15.35 km^2^ in 2006 because the new water areas that appeared in the spring of 2006 (depicted by the red pixels) appear to have connected the previously small Lake A to the larger lake system.

## Discussion

Temperature anomalies on Kanin have increased by approximately 1.4°C per decade since 1991. During the same period, there has been a marked trend of delays in herders’ arrivals to the Nes clinic. These changes are likely due to the lengthening of overall migration routes in order to avoid open water as well as to the necessity of waiting until later in the fall or early winter in order for the swampland and open water to freeze making the crossing possible for both humans and reindeer.

Over the last 3 decades, unusually warm autumns have progressively delayed the herders’ crossing of Kanin Neck on their way to winter pastures; their arrival at the Nes clinic has shifted from October to December (8) (Fig. 2). Concomitantly, until approximately 10 years ago, herders migrated later from winter pastures near the inland villages to Kanin Peninsula and arrived in Nes on April 6–10. In recent years, these spring arrivals to the clinic shifted to March 24–28 in order to avoid earlier thaws on the herders’ journey to points north (8). It appears that, in order to reach the summer calving areas before ice on Kanin Neck melts and renders the summer pastures unreachable, the herders shortened winter stays near the villages and stayed longer in Kanin, where they have virtually no access to non-emergency health care.

The study's findings corroborate our hypothesis that a relationship exists between the recently observed warming trend and the delays in the herders’ arrival at the Nes clinic. Formation of new lakes and streams, expansion of the existing water bodies, and a shorter winter (when these bodies of water are crossable over ice) all present formidable barriers to travel by foot, sled, and snowmobile. Spatial and temporal overlap of these environmental changes and their combined effect on transmigration may be responsible for the recent difficulties experienced by the herders in accessing primary and preventive care. There are several practical reasons for this.

The current increase and rapid fluctuation in the size and number of water bodies renders ground travel unpredictable because the herders and their herds must travel around the new water bodies and thus traverse greater distances in order to reach the clinic. For example, in 2006, in order to travel across the area shown in Fig. 5, the herders had to either wait until stable ice was formed over the lakes or deviate from their familiar historic routes and travel with large herds around the perimeter of the entire new water body (circled). Such deviations into unfamiliar terrain would increase total travel distance and might also increase the likelihood of injuries to humans and animals. The changes in the tundra hydrology reported here and their affect on livelihoods and access have been corroborated by the reindeer herders and local health care providers. Yet, if the current warming trend continues, it is not clear how these changes will affect the Nenets communities, such as Kanin Obshina, and other circumpolar peoples in the future. For example, do temperature anomalies and open water increase in a linear fashion or will they follow a different spatial pattern? More research is required to better understand the complex interrelationships between surface temperature increase, permafrost dynamics, shortened winter period, and increases in open water. Further development of scientifically sound methods for monitoring these changes at the community scale, as well as translating the data to be used by the community stakeholders and public health authorities, are critical steps in developing adaptation strategies in order to increase community resilience, advance healthcare delivery, and improve well-being in the changing circumpolar north.

## Conclusions

In this study, we used a novel approach to measure the relationship between temperature anomalies and access to a health care clinic by an indigenous population by combining clinician observations (arrival dates at the Nes clinic), CAM temperature anomalies, and MODIS-derived detection of open water. Until very recently, our ability to measure hydrologic change due to warming and to study its effects on to the health of Arctic peoples was severely limited by the dearth of high quality spatial and temporal data suitable for time-series analysis. Our novel use of clinic visit data, temperature records, and remotely sensed imagery permitted us to report on the recent interannual temporal shift in the herder families’ visits to the Nes health care clinic. It also demonstrated a relationship between the annual temperature increase, changes in surface water along the herder migration routes, and the capacity of the herders to sustain the levels of mobility needed for accessing viable pasture grounds for the reindeer while attending to their own health care needs. We further argue that this temporal shift is related to the warming temperatures on Kanin. However, similar direction of hydrologic changes has been reported in other regions of the circumpolar north that also have warmed to a comparably high level during the same period (2,17) and have negatively affected access to health care across the Arctic.

In conclusion, this investigation demonstrated the feasibility of future studies that rely on the participation and the local knowledge of the indigenous community and of local medical practitioners and on remotely sensed data. Given that researchers and local residents are observing similar changes in other parts of the circumpolar north, the approach demonstrated here is highly promising if applied to other Arctic contexts (for example, in connection with the coastal erosion and thermokarst processes and their implications for subsistence-based livelihoods and assess to health care).

Disparities in access to health care have been shown to negatively affect health outcomes elsewhere (17–20), and we suggest that new health care delivery models, responsive to the new reality of rapid Arctic environmental change, are necessary to improve the health of indigenous circumpolar peoples. One research direction with a potential to improve access to health care and to reduce the risk of injuries is to provide near-real-time new water detection maps directly to the subsistence-based circumpolar communities via an Internet portal. This approach may prove effective in visualizing environmental change and in planning future health services provision in the rapidly changing Arctic environment.
